# Medium- and time-related effects on hypothermic storage of rat testicular cells

**DOI:** 10.1530/RAF-22-0050

**Published:** 2023-06-08

**Authors:** Rageia Elfageih, Ahmed Reda, Kristín Rós Kjartansdóttir, Valentina Pampanini, Olle Söder, Jan-Bernd Stukenborg

**Affiliations:** 1 NORDFERTIL Research Lab Stockholm, Childhood Cancer Research Unit, Department of Women’s and Children’s Health, Karolinska Institutet, and Karolinska University Hospital, Solna, Sweden; 2 Danish National Genome Center, Ørestads Boulevard, København, Denmark; 3 Bambino Gesù Children’s Hospital, Piazza di Sant'Onofrio, Roma, Italia

**Keywords:** germ cells, sertoli cells, Leydig cells, mitochondria, fertility preservation

## Abstract

Testicular samples obtained for fertility preservation often need to be transported between clinics. This study aimed to mimic this short-term hypothermic storage (4–8°C) and explore the impact of these conditions and the transport medium composition on prepubertal rat testicular tissue samples. Testicular tissue samples obtained from 7 days *post-partum* rats were transferred to six compositionally different basal culture media and a balanced salt solution, which had been kept at 4–8°C prior to transfer. The samples were preserved for either 12 or 24 h in these hypothermic conditions. The potential effects of the short-term storage were evaluated by assessing the morphology, measuring the testosterone levels by radioimmunoassay and analysing 96 genes with TaqMan Low-Density Arrays. Levels of gene expression related to energy, apoptosis, and angiogenesis pathways were altered after hypothermic storage for 12 and especially 24 h. We observed only minor differences in gene expression profiles for germ and testicular somatic cells and no differences in tissue morphology and testosterone production levels. Short-term hypothermic storage of testicular tissue with a maximum duration of 24 h does not affect the overall expression profile of testicular cell-specific genes; however, in a minor way, it affects the expression of specific cellular genes.

**Lay summary:**

Male fertility depends on the proper functioning of cells which develop into reproductive cells. Due to an increasing number of childhood cancer survivors suffering from treatment-related fertility problems, as well as recent reports showing a dramatic decrease in sperm counts over the last decades, male fertility preservation has become an important research topic. To date, there is no method to restore fertility for men who are not able to produce sperm. One promising method to preserve the potential fertility of these patients is freezing tissue or cells from the testicles for future fertility treatments. A critical phase in freezing testicular tissue or cells is the time between removing the tissue from the testicles and freezing it. To better understand the impact of this phase on the quality of the testicular tissue, we used the testes of rats as a model for our research. We found that cooling testis tissue has only minor effects on the expression of genes that are important for testis function.

## Introduction

Male sub- and in- fertility is a growing modern-day problem which has been brought to light by confirmed decreasing sperm counts in adult males and reports on the negative effects of endocrine-disrupting compounds and medications on male fertility (Levine *et al.* 2017, Stukenborg *et al.* 2021). In particular, treatment-related fertility problems affecting the quality of life of childhood cancer survivors have led to an increasing number of prepubertal male patients participating in fertility preservation procedures worldwide (Goossens *et al.* 2020, Mulder *et al.* 2021). Various strategies have been attempted to offer a future treatment for sub- or infertile male patients to father biological children. Despite promising results in animal models, there is no established clinical tool available to restore fertility in these patients yet (Pampanini *et al.* 2020, Mulder *et al.* 2021). Consequently, cryopreservation protocols for testicular tissue or cells have been developed and explored to determine suitable storage conditions for testicular tissue samples for future use (Goossens *et al.* 2020).

Low temperatures decrease metabolic rates in tissue (Baust & Baust 2007, Guibert *et al.* 2011) and are intended to enhance cell viability and functionality (Yang & Honaramooz 2010). Transportation of testicular biopsies from the operating theater to the reproductive medicine unit where testicular cryopreservation and long-term storage occur is often an inevitability that could take up to 24 h and should therefore transpire in hypothermic conditions. Due to handling- and cost-related aspects, basal culture media such as Dulbecco’s modified Eagle’s medium/F12 (DMEM/12) or balanced salt solutions such as phosphate-buffered saline (PBS) are used for tissue sample transportation (Goossens *et al.* 2020). The increasing number of testicular tissue and cell samples stored for fertility preservation has led to an increasing number of studies, exploring the effect of hypothermic storage on testicular tissue and cells in different species (Jahnukainen *et al.* 2007, Zeng *et al.* 2009, Yang & Honaramooz 2010, Yang *et al.* 2010, Faes & Goossens 2016, 2017). Jahnukainen and colleagues (2007) investigated the effect of short-term cooling and cryopreservation on non-human primate spermatogonial cell survival and differentiation after xenotransplantation (Jahnukainen *et al.* 2007). The study revealed that testicular tissue samples stored for 24 h in ice-cold Leibovitz-L15 medium with 10% fetal calf serum showed the best results in graft recovery (after 3 months: 52%; after 5 months: 79%). In line with this finding, similar effects in porcine testicular tissue samples stored for 48 h at 4°C and subsequently xenografted into mice (Zeng *et al.* 2009), and comparable structural integrity, cell viability, *in vivo* growth of grafts, and germ cell development potential in tissue samples stored for 72 h at 4°C have been reported (Abrishami *et al.* 2010).

However, detailed studies on cellular mechanisms in testicular cells after hypothermic storage are still lacking. Therefore, this study addressed whether short-term hypothermic storage, as defined as preserving the testicular samples at 4–8°C for a maximum duration of 24 h and/or the composition of transport media affect tissue and cell morphology, testosterone production, and gene expression associated with fundamental cellular physiology of rat testicular cells.

## Materials and methods

### Animals and tissue preparation

Testes were obtained from 25 seven days *post-partum* (7d*pp*)-old Sprague Dawley rats, from different litters. The rats were purchased from Charles River (Sulzfeld, Germany) and transported to Karolinska Institutet (Stockholm, Sweden) together with their mothers. The experimental laboratory animal ethics committee approved the use and handling of animals at Karolinska Institutet (N489/11). After sacrifice, the testes were removed and immediately transferred to ice-cold DMEM, high glucose with pyruvate and l-Glutamine (P/N 41966, Gibco) with 1% penicillin/streptomycin (pen/strep; P/N 15070-022, Gibco). After de-capsulation, each testis was cut into three pieces (3–4 mm^3^ each), and each piece was placed into a 1.5 mL Eppendorf tube. The tubes were placed on ice and pre-filled with one of the six culture media or the balanced salt solution. The different media compositions used and supplemented with 1% pen/strep, included DMEM with glutamine (P/N 41966, Gibco), DMEM without glutamine (DMEM – glutamine; P/N 21969, Gibco), DMEM with Glutamax (P/N 31966, Gibco), DMEM/F12 (P/N 21331, Gibco), F12 (P/N 21765, Gibco), MEM (P/N 21430, Gibco), and PBS (P/N 14190, Gibco). The samples were either processed immediately as fresh tissue samples (0 h) or stored at 4–8°C for 12 or 24 h. For each culture medium used, three testicular tissue samples obtained from different animals of different litters were collected at three time points: 0, 12, and 24 h (a total of three samples per medium per time point and analysis).

### Embedding and sectioning

Samples for histological evaluation were fixed in 4% paraformaldehyde (P/N 8187081000, Merck) at 4°C overnight. The samples were dehydrated in ascending ethanol series (30, 50, and 70%) at room temperature (RT) for a duration of 24 h each. Further tissue dehydration proceeded in 80, 96, and 99.6% ethanol at RT (for 6 h each). Clearance of ethanol proceeded in butyl acetate (P/N 45860, Sigma Aldrich) for 6 h at RT. Samples were transferred to liquefied paraffin (Paraplast X-TRA®; P/N P3808, Sigma Aldrich) and kept at 61°C overnight. After moulding, blocks were placed at −20°C for 30 min to solidify. A microtome (Reichert-Jung, Depew, NY, USA) was used to section the samples at 5 µm thickness. Sections were placed on glass slides (P/N J1800AMNZ, Superfrost Plus, Thermo Scientific) and placed vertically overnight at 37°C.

### Morphological evaluation and periodic acid-Schiff (PAS) staining

The tissue sections were de-paraffinized with xylene for 10 min, then gradually rehydrated with 99.6, 96, and 70% ethanol and washed twice with 1x PBS (pH 7.4, P/N 14190-094, Gibco). Each of the rehydration steps was performed twice for 5 min. After rehydration, the sections were washed in distilled water twice for 5 min and incubated in periodic acid for 5 min (PAS kit (101646, Merck)). This step was followed by thorough washing under running tap water and then twice in distilled water for 5 min each. Sections were then incubated in Schiff’s reagent for 15 min (PAS kit (101646, Merck)) and washed thoroughly under running tap water and twice in distilled water for 5 min each. Samples were counterstained with hematoxylin (Mayer’s hemalum solution, 1092491000, Merck) for 2 min and washed under running tap water for 2 min. Slides were dehydrated in ascending ethanol concentrations and xylene and mounted with Entellan® new (P/N 1079610100, Merck). All samples were analyzed under an Eclipse E800 microscope (Nikon), and pictures were taken with a 12.5 million-pixel cooled digital color camera system (Olympus DP70).

### Testosterone radioimmunoassay (RIA)

Leydig cell functionality was assessed by testosterone RIA. After storage for 24 h in hypothermic conditions, testicular tissue samples were cultured in DMEM with glutamine, supplemented with 5 IU/L human chorionic gonadotropin (hCG) and 5 IU/L recombinant follicle-stimulating hormone (FSH) at 34.5°ºC and 5% CO_2_ for 24 h. Testicular tissue weights were measured for each sample (weight per tissue sample: 14 ± 3.9 mg), and 200 µL 1x PBS was added to each sample. To extract the testosterone, samples were sonicated using Vibra Cell™ (Sonics and Materials Inc., Newtown, CT, USA) for 30 s. All samples received ethyl acetate (P/N 300612, Merck) before they were subjected to shaking for 15 min on an automatic shaker at 1000 rpm (IKA-VIBRAX-VXR, Staufen, Germany). After 2 min of centrifugation at 16,000 ***g***, the supernatant was transferred into a new Eppendorf tube, and the process was repeated once more to ensure complete extraction. The ethyl acetate in the collected supernatant of each tube was evaporated overnight, and the pellets containing testosterone were dissolved in 1x PBS and COAT-A-COUNT® RIA kit (P/N TKTT2, Siemens) was used to evaluate testosterone according to the manufacturer’s protocol. In brief, extracted samples were placed in the specified tubes, supplemented with I^125^ total testosterone (supplied with the kit), and incubated in a water bath at 37°C for 3 h while shaking at 120 rpm. Then, solutions were decanted, and the radioactivity in the tubes was measured by a Gamma counter (1470 Wizard Wallac, GMI, MA, USA) for 1 min. The intra-testicular testosterone production was measured in nanogram testosterone per milligram testis weight. Standards (0–55 nmol/L) provided with the kit were used for calibration.

### RNA extraction and cDNA synthesis

For RNA extraction, the RNeasy Mini Kit (P/N 74104, Qiagen) and homogenization by an ULTRA-TURRAX T25 homogenizer (JANKE & KUNKEL, Staufen, Germany) twice for 30 s were used according to the manufacturer’s protocol. To prevent DNA contamination, isolated RNA samples were treated with DNase I Amplification Grade (P/N AMPD1, Sigma-Aldrich). IScript™ cDNA synthesis kit (P/N 170-8891, Bio-Rad) was used to synthesize cDNA from the RNA samples according to the manufacturer's protocol.

### Reverse transcriptase-polymerase chain reaction (RT-PCR)

As endogenous control to test the cDNA quality, β-actin (*Actb (*Forward primer: TGA AGA TCA AGA TCA TTG CTC C; Reverse primer: ACT CAT CGT ACT CCT GCT TGC) was used. RT-PCR was performed with the Expand High Fidelity PCR System (P/N 11759078001, Roche) using 2720 Thermal Cycler (Applied Biosystems, Life Technologies). The cycling phases of the RT-PCR were the DNA template denaturation phase at 96°C for 30 s, the primer annealing phase at 58°C for 30 s, and the primer extension phase at 72°C for 1 min. The cycling phases were repeated for 35 cycles; the samples were kept in the final extension phase at 72°C for 5 min, followed by a cooling phase at 4°C.

### TaqMan® low-density array (TLDA)

The prepubertal rat testicular samples were stored in cold media with different compositions and collected at 0, 12, and 24 h. RNA was extracted from the collected samples as described earlier. TLDA cards were custom-designed to analyze the gene expression profiles of testicular cell types and included 96 different genes, covering those expressed in germ cells (*n* = 27), Sertoli cells (*n* = 9), Leydig cells (*n* = 8), as well as angiogenesis- (*n* = 16), proliferation- (*n* = 8), apoptosis- (*n* = 4), and energy (*n* = 18) genes. The endogenous control genes (*n* = 6) were used for normalization. Each sample was loaded in triplicate onto the cards. Cards were purchased from Applied Biosystems, Life Technologies. Details of the TaqMan® probes for detecting the different genes and their assay numbers are all listed in Supplementary Table 1.

### Gene Ontology (GO) enrichment analysis

The GO enrichment analysis (Panther17.0 released, Gene ontology consortium) (Mi *et al.* 2019) was used to analyze the obtained TLDA card data and identify genes expression levels that displayed significant change with a *P*-value less than 0.05 (*P* < 0.05) (reference list: *Rattus norvegicus*). The analysis was performed according to instructions provided by the GO consortium online (http://geneontology.org; DOI: 10.5281/zenodo.5725227 Released 2020-11-01). PANTHER Overrepresentation Test (Released 20221013) was additionally performed.

### Statistical analysis

The relative fold-change of gene expression in each medium at the three-storage time points (0, 12, and 24 h) was calculated using the delta-delta Ct method (*ddCt*). The six housekeeping genes were used to calculate the delta Ct (*dCt*). The average *dCt* of the fresh tissue control samples (0 h) was calculated and used to normalize the *dCt* values to obtain the *ddCt* of each gene at three time points (0, 12, and 24 h). The fold-change of each gene was calculated using power (2^−^*^ddCt^*). One-way ANOVA (non-parametric, mixed) analysis was used to determine the significant fold-changes in gene expression profile at three storage time points (0, 12, and 24 h) and between different media. The fold-change of each gene (mean ± S.D.) was plotted against three storage time points and against various culture media at three-storage time points. The mean differences were considered significant when the *P*-value was less than 0.05 (*P* < 0.05). All statistical analyses were performed using GraphPad Prism version 9.3. (GraphPad Software).

## Results

In this study, we tested the effect of hypothermic conditions (4–8°C) and storage time (12 and 24 h) on the quality of prepubertal rat testicular tissue preserved in basal culture media and balanced salt solution (1× PBS). The basal cell culture media were either DMEM without or with glutamine, Glutamax, or F12; in addition, MEM and F12 culture media were included. The time points correspond to the estimated time required to transport testicular tissue biopsy samples from one hospital to another to perform the cryopreservation.

### Medium composition or storage time in hypothermic conditions does not affect cell morphology or Leydig cell functionality

PAS staining was performed to assess the morphological status of the prepubertal rat testicular tissue in different culture media at the two time points. We observed no morphological differences when comparing the rat prepubertal testicular samples collected at both time points to fresh samples, regardless of the media used to store the samples (Supplementary Figs. 1 and 2, respectively, see section on supplementary materials given at the end of this article). In line with previously published studies (Yang & Honaramooz 2010, Faes & Goossens 2016), our evaluation showed morphologically intact gonocytes and spermatogonia at both time points in all conditions. Furthermore, no negative effects on interstitial or intratubular somatic cells could be observed. No significant differences in testosterone production levels in tissue samples stored for 24 h in different cold culture media or 1x PBS could be observed after stimulation with FSH and hCG for 24 h *in vitro* (Supplementary Fig. 3).

### The effect of storage time on gene expression levels in germ and testicular somatic cells

To explore the effect of the storage time in different culture media, custom-designed TLDA cards were used to investigate the gene expression profile of 96 genes related to germ cells, testicular somatic cells (e.g. Leydig, Sertoli, and peritubular myoid cells and macrophages), proliferation, various cellular pathways, such as apoptosis, angiogenesis, energy, and six housekeeping genes (*18S*, *Actb*, *B2m*, *Ctnnb1*, *Gapdh*, and *Eef1a1*) (Supplementary Table 1). Samples that exhibited time-related changes in gene expression profiles due to hypothermic storage did not show significant media-related effects on cell- and pathway-specific gene expression (Supplementary Figs. 4, 5, 6, 7, 8, 9, and 10). Significant differences were observed in samples stored for 12 and 24 h compared to fresh tissue control samples (0 h). Germ cell-related genes, such as *Gfra-1* and *Cd9*, showed significant downregulation within 24 h of storage, while *Kit* showed significant upregulation. Furthermore, *Zbtb16* expression was significantly downregulated in 12 h; however, it retained a normal level after 24 h (Supplementary Fig. 11). On the other hand, non-significant changes were observed in the expression profile of *Crem, Csf1, Dnmt3b, Fgf5, Fgfr3, Fut4, Gdf3, Gdnf, Lefty2, Nodal, Plaa, Pou5f1, Scp3, Tdgf1, Tfap2c,* and *Thy1* compared to fresh tissue control samples.

Genes expressed in Sertoli cells showed significant downregulation of *Wt1*, *Kitlg*, *Vim, and Fshr* genes compared to fresh tissue control samples (0 h) after 12 and/or 24 h. However, *Amh, Ar, Gata-4, Gata-6, Inhbb,* and *Sox9* showed no significant changes at both storage time points compared to the fresh tissue control samples. Moreover, genes expressed in Leydig cells showed significant downregulation of *Lhcgr* and *Tspo* genes or upregulation of *Star* gene at 24 h compared to the control (Supplementary Fig. 12). No significant changes were observed in the gene expressions of *Cyp-17, Cyp-11, Hsd3b, and Insl3* compared to fresh tissue control samples.

Taken together, although statistically significant, the changes in gene expression do not reflect major biological changes that can be detected in the morphological status of the germ and somatic cells or functionality of Leydig cells that are indicated by normal testosterone levels, as mentioned earlier.

### The effect of storage time on gene expression profiles of proliferation, angiogenesis, energy, and apoptosis-related genes might reflect the cellular status of the testicular cells

**Figure 1:**
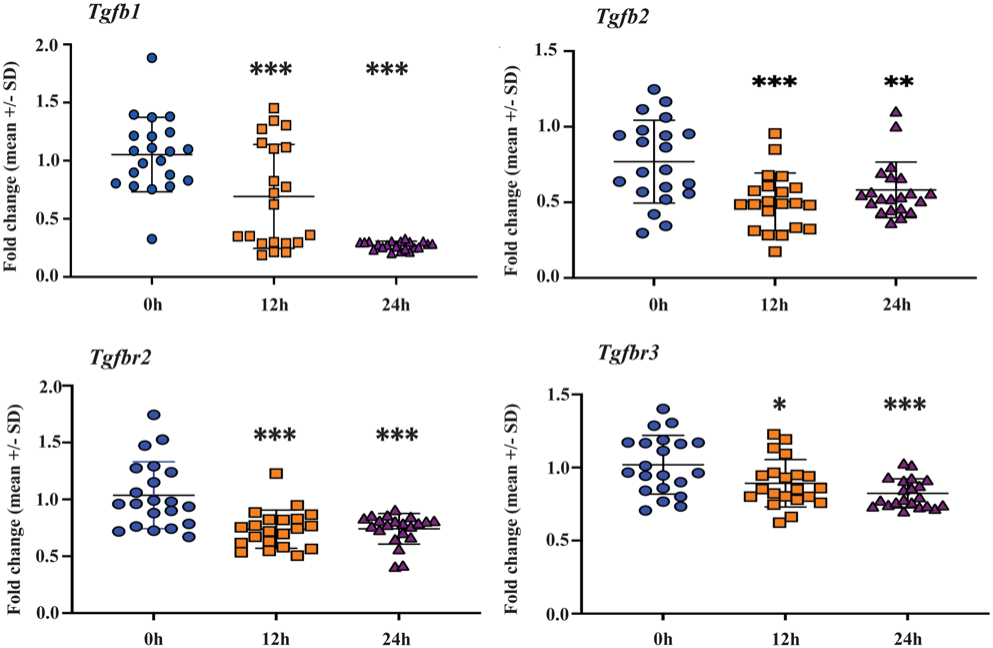
TaqMan low-density array (TLDA) analysis of genes related to angiogenesis. Transforming growth factor beta 1 (*Tgfb1*), transforming growth factor beta 2 (*Tgfb2*), transforming growth factor beta receptor type 2 (*Tgfbr2*), and transforming growth factor beta receptor type 3 (*Tgfbr3*) were significantly downregulated after 12 and 24 h storage time at hypothermic condition when the fold-change expression values were compared to fresh tissue control samples at 0 h storage time. The mean ± s.d. of each gene at three time points is *Tgfb1*: 0 h (1.05 ± 0.32), 12 h (0.69 ± 0.44), 24 h (0.27 ± 0.03); *Tgfb2*: 0 h (0.76 ± 0.27), 12 h (0.50 ± 0.18), 24 h (0.58 ± 0.18); *Tgfbr2*: 0 h (1.03 ± 0.29), 12 h (0.73 ± 0.16), 24 h (0.74 ± 0.13); and *Tgfbr3*: 0 h (1.01 ± 0.20), 12 h (0.89 ± 0.16), 24 h (0.82 ± 0.09). The number of samples per each group is *n* = 21, and the *P*-values are as follows: *P < 0.05, **P < 0.01, ***P < 0.001.

After 24 h in hypothermic conditions, downregulation of genes connected to proliferation (*Top2a*, *Ccnd1*, and *Tk1, Mki67*, *Cdkn1*a) was observed. However, a significant upregulation of *Cdk1b* expression was measured at 24 h (Supplementary Fig. 13). *Tgfb1, Tgfb2,Tgfbr2*, and *Tgfbr3,* which are related to inflammation, showed significant downregulation (*Tgfb1*, *Tgbfr2, Tgfb2* at 12 h, and *Tgfbr3* at 24 h), in both storage time points compared to the fresh tissue control samples (Fig. 1). While a significant downregulation in the expression profile of *Kdr* was observed at 24 h, *Pecam1*, *Fit*, *Fgf1*, *Pdgfra, Pdgfrb*, and *Fgf2* were significantly downregulated already after 12 h (Fig. 1, and Supplementary Fig. 14).

**Figure 2:**
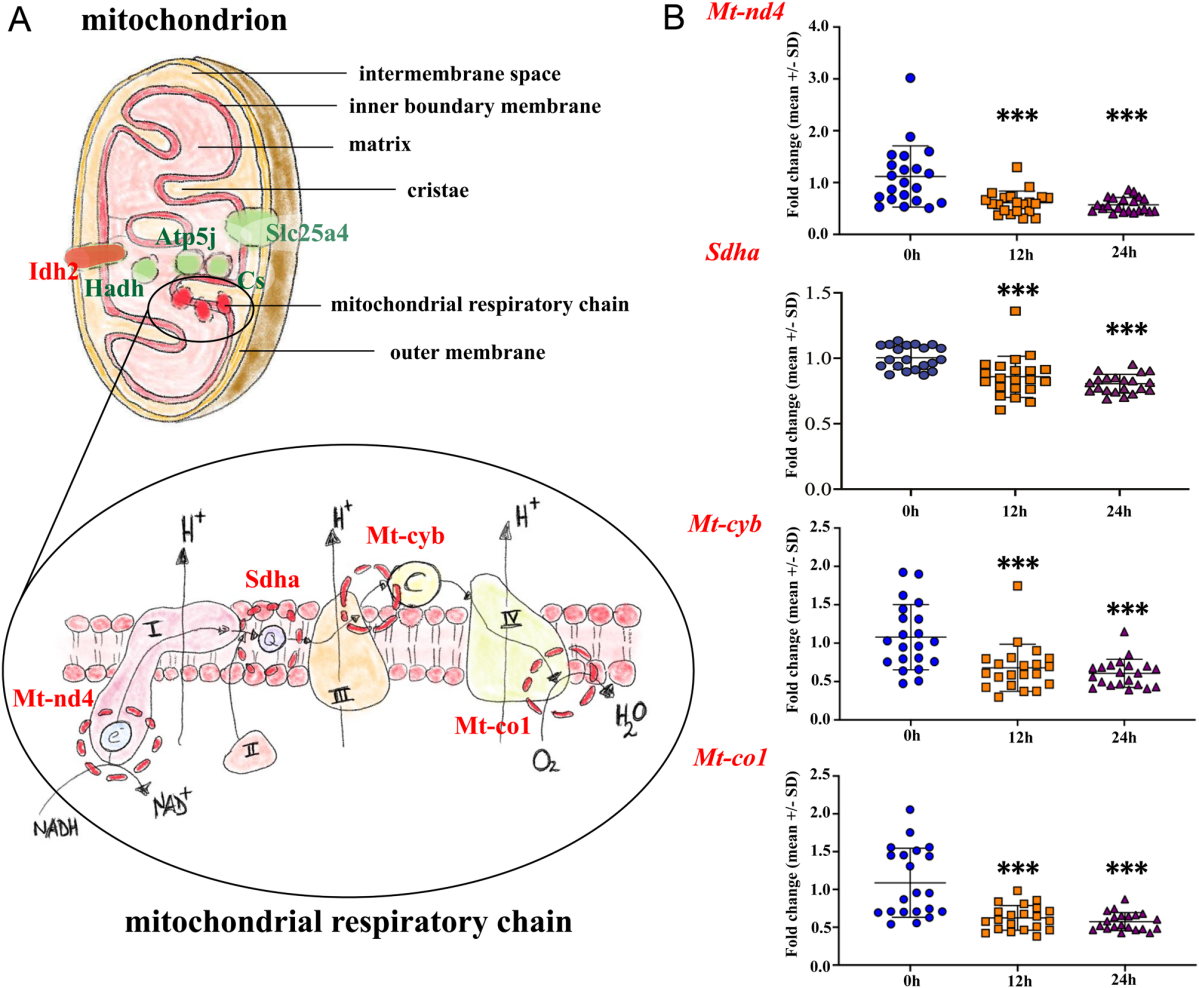
TaqMan low-density array (TLDA) analysis of genes related to the energy. (A) The schematic overview of proteins encoded for genes affected (red color) and non-affected (green color) in mitochondria and the energy was evaluated by TLDA-card analysis. (B) Significantly lower expression of mitochondrially encoded NADH:ubiquinone oxidoreductase core subunit 4 (*Mt-nd4*), mitochondrially encoded cytochrome B (*Mt-cyb*), mitochondrially encoded cytochrome C oxidase I (*Mt-co-1*), and succinate dehydrogenase A (*Sdha*) after 12 and 24 h storage time at hypothermic condition compared to fresh tissue controls. The mean ± S.D. of each gene at three time points is *Mt-nd4*: 0 h (1.11 ± 0.59), 12 h (0.61 ± 0.22), 24 h (0.57 ± 0.14); *Mt-cyb*: 0 h (1.07 ± 0.42), 12 h (0.67 ± 0.30), 24 h (0.60 ± 0.18); *Mt-co-1:* 0 h (1.08 ± 0.45), 12 h (0.62 ± 0.16), 24 h (0.57 ± 0.11); *Sdha:* 0 h (1.03 ± 0.29), 12 h (0.73 ± 0.16), 24 h (0.74 ± 0.13). The number of samples per each group is *n* = 21, and the *P-*values are as follows: *P < 0.05, **P < 0.01, ***P < 0.001.

Most genes related to energy pathways were downregulated; in particular, the genes related to the energy in mitochondria (*Mt-nd4*, *Mt-co1*, *Sdha*, and *Mt-cyb)* exhibited downregulation at both storage time points compared to the fresh tissue control samples (Fig. 2). Moreover, *Hif1-a* and *Prkaa1* genes showed significant downregulation in both time points compared to fresh tissue control samples. In addition, *Ihd2* and *Ldhb* displayed a significant downregulation at 24 h of storage time compared to fresh tissue control samples. However, downregulation of *Slc2a4*, the gene that encodes solute carrier family 2 (facilitated glucose transporter) member 4, was observed at 12 and 24 h (Supplementary Fig. 15).

**Figure 3:**
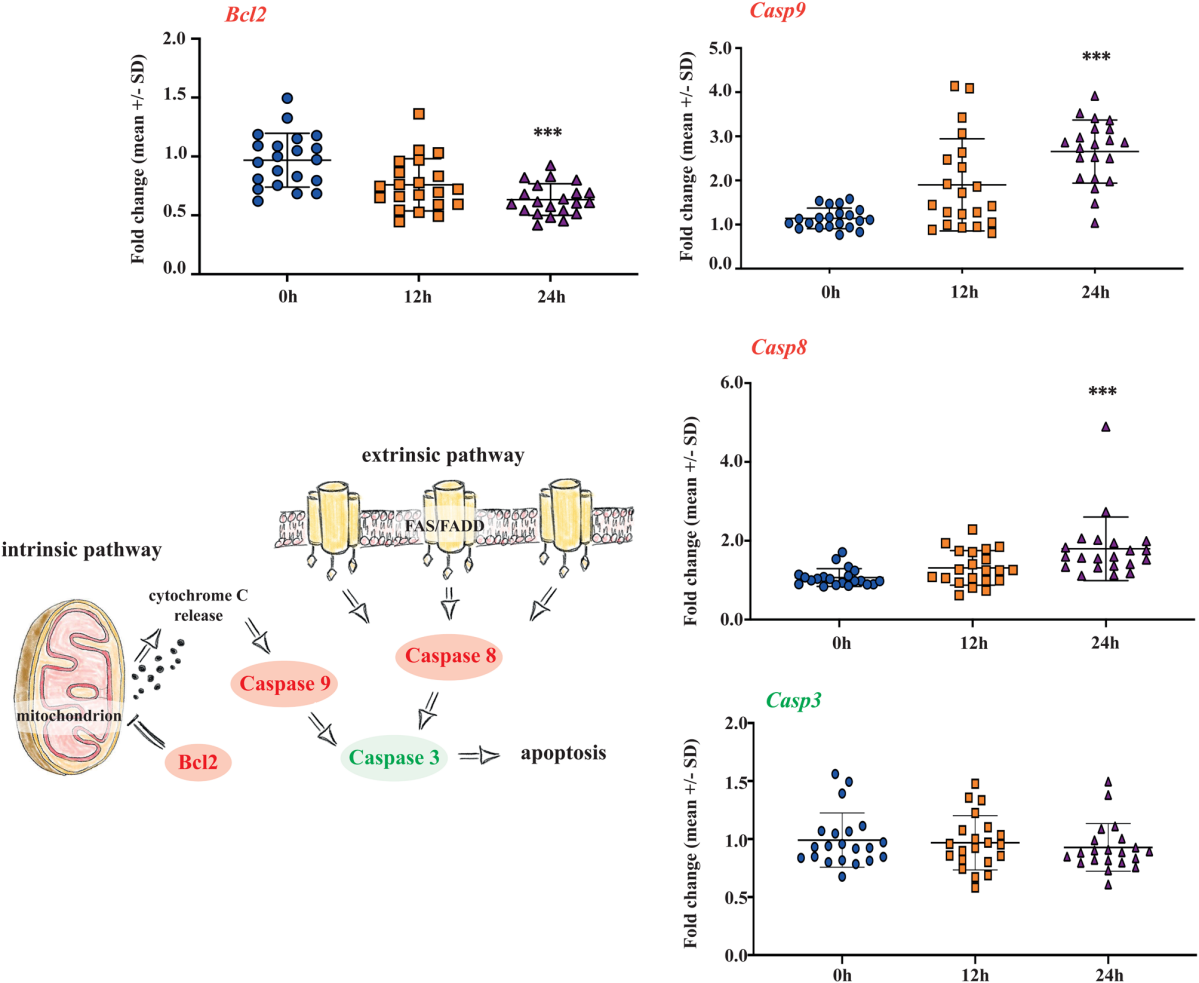
TaqMan low-density array (TLDA) analysis of genes related to intrinsic and extrinsic apoptosis pathways. A significant upregulation of Caspase 8 (*Casp8*) and Caspase 9 (*Casp9*); and downregulation of apoptosis regulator B-cell lymphoma 2 (*Bcl2*) was observed at 24 h storage time at the hypothermic condition when the fold-change expression values were compared to fresh tissue controls. However, significant changes were not observed in Caspase 3 (*Casp3*) gene expression after short-term hypothermic storage when the fold-change expression values were compared to fresh tissue controls. The mean ± S.D. of each gene at three time points is *Casp8*: 0 h (1.06 ± 0.22), 12 h (1.31 ± 0.43), 24 h (1.79 ± 0.80); *Casp9*: 0 h (1.13 ± 0.23), 12 h (1.89 ± 1.04), 24 h (2.65 ± 0.71); *Bcl2:* 0 h (0.96 ± 0.22), 12 h (0.75 ± 0.22), 24 h (0.63 ± 0.13); *Casp3:* 0 h (0.99 ± 0.23), 12 h (0.96 ± 0.23), 24 h (0.92 ± 0.20). The number of samples per each group is *n* = 21, and the *P-*values are as follows: *P < 0.05, **P < 0.01, ***P < 0.001.

The testicular tissue samples that had been stored for 24 h in hypothermic conditions, exhibited a significant upregulation of the apoptosis-related genes *Casp8* (member of the intrinsic apoptosis pathway) and *Casp9* (member of the extrinsic apoptosis pathway), and significant downregulation of *Bcl2* (encoding for the apoptosis-inhibiting protein BCL2 located in the mitochondrial membrane). However, no change was observed in the *Casp3* (final member of both intrinsic and extrinsic apoptosis pathway) gene expression profile (Fig. 3). GO enrichment analysis revealed that the most affected biological, cellular, and molecular functions by hypothermic storage were correlated to TGFb signaling pathway, protein phosphorylation, receptor complexes, growth factor binding, cytokine receptor binding, and activation of apoptosis-related cysteine-type endopeptidase activity (Supplementary Table 2). Pathways identified for upregulated genes were related to FAS and apoptosis signaling pathways, while genes showing a downregulation were related to angiogenesis pathways (Supplementary Table 2).

## Discussion

This study focused on the effect of different basal cell culture medium compositions and hypothermic conditions on prepubertal rat testicular tissues stored at 12 and 24 h to mimic the estimated time required to transport testicular biopsies to cryopreservation facilities. In agreement with previous studies performed on different species (Jahnukainen *et al.* 2007, Yang & Honaramooz 2010, Faes & Goossens 2016), no medium- and storage-related morphological and histological changes could be observed. Furthermore, no impaired ability to produce testosterone after stimulation with FSH and hCG of the stored testicular tissue samples was detected.

Although previously published studies showed high survival rates and germ cell differentiation potential in transplanted tissue samples with an equal or slightly better performance when compared to fresh tissue control samples (Jahnukainen *et al.* 2007, Zeng *et al.* 2009, Abrishami *et al.* 2010), a detailed study on gene expression profiles in these tissue samples has not yet been conducted. Therefore, we focused on the effects of different basal cell culture media, hypothermic conditions, and storage times on a fundamental gene expression profile of testicular tissue. Observed changes in the gene expression profile of germ cells, somatic cells, and genes related to cellular processes, such as apoptosis, angiogenesis, and energy pathways, could not be related to any specific basal cell culture medium composition or hypothermic conditions. However, storage time affects the gene expression profile of some genes related to germ and somatic cells and fundamental cellular processes. An altered expression of genes expressed in undifferentiated spermatogonia could be observed at both storage times; however, other germ-cell-related genes, expressed in spermatocytes and spermatids, did not reveal any significant changes in expression profiles and did not seem affected by the storage.

The effect of storage time also altered the gene expression profile of testicular somatic cells. Genes expressed in Sertoli cells showed downregulated gene expression after 12 h (*Wt1*) and 24 h (*Vim*, *Kitlg*, and *Fshr*), while genes expressed in Leydig cells genes showed both downregulation (*Lhcgr* and *Tspo*) and upregulation (*Star*) after 24 h of storage. Although significant changes in gene expression profiles of certain germ and somatic cells, connected to undifferentiated germ cells and their niche, might suggest a potential effect on SSCs, the relatively short storage time might not impact the overall functionality or quality of the tissue in the long term. This is in line with previously published reports (Yang & Honaramooz 2010, Faes & Goossens 2016, 2017) and further supported in this study by a lack of differences in the testicular tissue morphology, as well as testosterone production when comparing the tissue samples stored in different culture media at hypothermic conditions.

Focusing on expression profiles of genes connected to fundamental biological processes, a downregulation of proliferation genes, *Top2a*, *Mki67*, *Ccnd1*, *Cdkn1*a and *Tk1,* and an upregulation of *Cdk1* could be observed after hypothermic storage for 24 h.

After 12 h in hypothermic conditions, the profile of energy-related genes showed a downregulation of 9 out of 18 genes. Genes related to the energy complex (*Mt-nd4, Mt-co1, Mt-cyb,* and *Sdha*) and the mitochondrial ADP/ATP translocator (*Slc25a4*), exhibited a significant downregulation. An accumulation of oxygen radicals with time in the mitochondria can damage the mitochondrial cell membrane via lipid peroxidation reactions (Guo *et al.* 2013). This might be related to the upregulation of apoptosis-related genes *Casp8* and *Casp9*, and the downregulation of *Bcl2*, an apoptosis inhibitor seen after 24 h of storage in our study. Upon mitochondrial membrane lipid peroxidation, cytochrome C is released to the cytoplasm and activates the caspase cascade, which leads to apoptosis (Garrido *et al.* 2006). However, in our study, the expression of the *Casp3* gene did not change, which might indicate that the short-term effect of the storage time will not operate the whole cascade of apoptosis. A previous study has shown that the hypoxic state of the tissue can induce downregulation of the *Tgfb1* (Hadjipanayi & Schilling 2013, Zhou *et al.* 2018). TGFb1 has anti-apoptotic properties that suppress the apoptosis cascades, which might explain the upregulation of *Casp8* and *Casp9* (Chen *et al.* 2003). Our GO analysis has shown changes in cysteine-type endopeptidases, that are involved in the execution phase of apoptosis, its process and signaling pathway.

In conclusion, this study suggests that using basal cell culture media or balanced salt solutions to transfer and preserve the testicular tissue in hypothermic conditions for up to 24 h does not affect the morphology or functionality of the stored tissue. However, further studies are required to investigate whether the gene expression changes, particularly the angiogenesis, energy, and apoptosis-related genes observed in this study in rats, are similar in humans. Moreover, monitoring these genes before and after cryopreservation might be useful to ensure the best tissue quality for future use in cell and tissue transplantation assays and *in vitro* culture conditions intended to rescue fertility in boys and men suffering from fertility problems.

## Supplementary Materials

Supplementary Table 1. The genes used in TaqMan Low-Density Array (TLDA) analysis.

Supplementary Table 2. The Gene Ontology enrichment analysis after Bonferroni correction (Panther17.0 released, Gene ontology consortium) which focused on biological processes, cellular components, molecular functions and pathways in rats. Significantly up- and downregulated compared to the controls. Data were obtained with TaqMan low-density array card data analysis (P-value < 0.05).

Supplementary Figure 1

Supplementary Figure 2

Supplementary Figure 3

Supplementary Figure 4

Supplementary Figure 5

Supplementary Figure 6

Supplementary Figure 7

Supplementary Figure 8

Supplementary Figure 9

Supplementary Figure 10

Supplementary Figure 11

Supplementary Figure 12

Supplementary Figure 13

Supplementary Figure 14

Supplementary Figure 15

## Declaration of interest

The authors declare that there is no conflict of interest that could be perceived as prejudicing theimpartiality of the research reported..

## Funding

The project was financed by grants provided by the Jane and Dan Olssons Foundation, Frimurare Barnhuset i Stockholm, Kronprinsessan Lovisas Förening För Barnasjukvård/Stiftelsen Axel Tielmans Minnesfond, the Samariten Foundation, the Swedish Childhood Cancer Foundation (TJ2020-0023) and the Birgitta and Carl-Axel Rydbeck’s Research Grant for Paediatric Research (2022-00317). RE was supported by the Finnish Cancer Society.

## Author contribution statement

RE, AR, and J-BS conceived and designed the experiments and interpreted the results. AR, KRK, VP carried out data collection; RE and AR performed the statistical analyses; J-BS, AR, RE, KRK, VP, and OS analyzed the data; RE, AR, and J-BS wrote the initial draft of the manuscript. All authors commented, edited and approved the final version of the manuscript.
