# Supplementary material for: Medium- and time-related effects on hypothermic storage of rat testicular cells
Source: Reprod Fertil. 2023 Jun 8;4(2):e220050. doi: 10.1530/RAF-22-0050 (PMC10305459; doi:10.1530/RAF-22-0050)
Supplement: Supplementary Table 1. The genes used in TaqMan Low-Density Array (TLDA) analysis. [file supplementary_table_1.pdf]

Supplementary table 1

Number	Group	Gene name - Assay
1	Angiogenesis	***Angpt1*-Rn00585552_m1**
2	Angiogenesis	***Angpt2*-Rn01756774_m1**
3	Angiogenesis	***Cdh5*-Rn01536708_m1**
4	Angiogenesis	***Emcn*-Rn01521919_m1**
5	Angiogenesis	***Fgf1*-Rn00689153_m1**
6	Angiogenesis	***Fgf2*-Rn00570809_m1**
7	Angiogenesis	***Flt1*-Rn00570815_m1**
8	Angiogenesis	***Kdr*-Rn00564986_m1**
9	Angiogenesis	***Pdgfra*-Rn01399472_m1**
10	Angiogenesis	***Pdgfrb*-Rn00709573_m1**
11	Angiogenesis	***Pecam1*-Rn01467262_m1**
12	Angiogenesis	***Tgfb1*-Rn00572010_m1**
13	Angiogenesis	***Tgfb2*-Rn00579674_m1**
14	Angiogenesis	***Tgfbr2*-Rn00579682_m1**
15	Angiogenesis	***Tgfbr3*-Rn00568482_m1**
16	Angiogenesis	***Vegfa*-Rn01511601_m1**
17	Apoptosis	***Bcl2*-Rn99999125_m1**
18	Apoptosis	***Casp3*-Rn00563902_m1**
19	Apoptosis	***Casp8*-Rn00574069_m1**
20	Apoptosis	***Casp9*-Rn00581212_m1**
21	Energy	***Atp1a1*-Rn01533986_m1**
22	Energy	***Atp5j*-Rn00821491_g1**
23	Energy	***Cs*-Rn00756225_m1**
24	Energy	***Eif2ak3*-Rn00581002_m1**
25	Energy	***Hadh*-Rn00589352_m1**
26	Energy	***Hif1a*-Rn01472827_g1**
27	Energy	***Idh2*-Rn01478119_m1**
28	Energy	***Ldha*-Rn00820751_g1**
29	Energy	***Ldhb*-Rn00754927_m1**
30	Energy	***Ldhc*-Rn00568562_m1**
31	Energy	***MT-CO1*-Rn03296721_s1**
32	Energy	***MT-CYB*-Rn03296746_s1**
33	Energy	***MT-ND4*-Rn03296781_s1**
34	Energy	***Prkaa1*-Rn00665045_m1**
35	Energy	***Sdha*-Rn00590475_m1**
36	Energy	***Slc25a4*-Rn00821477_g1**
37	Energy	***Slc2a1*-Rn01417099_m1**
38	Energy	***Slc2a4*-Rn01752377_m1**
39	Germ cell	***Acr*-Rn00560621_m1**

40	Germ cell	***Cd9*-Rn01463253_m1**
41	Germ cell	***Crem*-Rn01538528_m1**
42	Germ cell	***Csf1*-Rn00696122_m1**
43	Germ cell	***Dnmt3b*-Rn01536419_m1**
44	Germ cell	***Fgf4*-Rn00709728_m1**
45	Germ cell	***Fgf5*-Rn00573575_m1**
46	Germ cell	***Fgfr3*-Rn00584799_m1**
47	Germ cell	***Fut4*-Rn00573633_s1**
48	Germ cell	***Gdf3*-Rn01492304_m1**
49	Germ cell	***Gdnf*-Rn00569510_m1**
50	Germ cell	***Gfra1*-Rn01444617_m1**
51	Germ cell	***Kit*-Rn00573942_m1**
52	Germ cell	***Lefty1*-Rn01412531_g1**
53	Germ cell	***Lefty2*-Rn02345859_m1**
54	Germ cell	***LOC100362033*;*Sycp3*-Rn00564846_m1**
55	Germ cell	***Nanog*-Rn01462825_m1**
56	Germ cell	***Nodal*-Rn01433624_m1**
57	Germ cell	***Plaa*-Rn00587888_m1**
58	Germ cell	***Pou5f1*-Rn01532129_g1**
59	Germ cell	***Prm1*-Rn02345725_g1**
60	Germ cell	***Prm2*-Rn00563507_g1**
61	Germ cell	***Tdgf1*-Rn01445056_g1**
62	Germ cell	***Tfap2c*-Rn01528879_m1**
63	Germ cell	***Thy1*-Rn00562048_m1**
64	Germ cell	***Zbtb16*-Rn01418644_m1**
65	Housekeeping	***18S*-Hs99999901_s1**
66	Housekeeping	***Actb*-Rn00667869_m1**
67	Housekeeping	***B2m*-Rn00560865_m1**
68	Housekeeping	***Ctnnb1*-Rn00584431_g1**
69	Housekeeping	***Gapdh*-Rn01749022_g1**
70	Housekeeping	***LOC100360413*;*Eef1a1*-Rn01639851_g1**
71	Leydig cell	***Cyp11a1*-Rn00568733_m1**
72	Leydig cell	***Cyp17a1*-Rn00562601_m1**
73	Leydig cell	***Cyp19a1*-Rn01422546_m1**
74	Leydig cell	***Hsd3b*-Rn01789220_m1**
75	Leydig cell	***Insl3*-Rn00586632_m1**
76	Leydig cell	***Lhcgr*-Rn00564309_m1**
77	Leydig cell	***Star*-Rn00580695_m1**
78	Leydig cell	***Tspo*-Rn00560892_m1**
79	Proliferation	***Ccnd1*-Rn00432360_m1**

80	Proliferation	***Ccne1*-Rn01457762_m1**
81	Proliferation	***Cdkn1a*-Rn01427989_s1**
82	Proliferation	***Cdkn1b*-Rn00582195_m1**
83	Proliferation	***Mki67*-Rn01451446_m1**
84	Proliferation	***Pcna*-Rn01514538_g1**
85	Proliferation	***Tk1*-Rn01456339_g1**
86	Proliferation	***Top2a*-Rn00573347_m1**
87	Sertoli cell	***Amh*-Rn00563731_g1**
88	Sertoli cell	***Ar*-Rn00560747_m1**
89	Sertoli cell	***Fshr*-Rn01648507_m1**
90	Sertoli cell	***Gata4*-Rn01530459_m1**
91	Sertoli cell	***Gata6*-Rn00569873_m1**
92	Sertoli cell	***Inhbb*-Rn01753772_m1**
93	Sertoli cell	***Kitlg*-Rn01502851_m1**
94	Sertoli cell	***Sox9*-Rn01751069_mH**
95	Sertoli cell	***Vim*-Rn00579738_m1**
96	Sertoli cell	***Wt1*-Rn00580566_m1**
