# Supplementary material for: Medium- and time-related effects on hypothermic storage of rat testicular cells
Source: Reprod Fertil. 2023 Jun 8;4(2):e220050. doi: 10.1530/RAF-22-0050 (PMC10305459; doi:10.1530/RAF-22-0050)
Supplement: Supplementary Table 2. The Gene Ontology enrichment analysis after Bonferroni correction (Panther17.0 released, Gene ontology consortium) which focused on biological processes, cellular components, molecular functions and pathways in rats. Significantly up- and downregulated compared to the controls [file supplementary_table_2.pdf]

Supplementary table 2

**PANTHER GO-Slim Biological Process** **(Genes upregulated after 24 hours)**	***Rattus norvegicus* (REF)**	**Fold-enrichment**	**+/-**	**P value**
activation of cysteine-type endopeptidase activity involved in apoptotic process	13	4	+	$4.97*10^{-3}$
↳positive regulation of cysteine-type endopeptidase activity involved in apoptotic process	21	> 100	+	$1.20*10^{-2}$
↳positive regulation of cysteine-type endopeptidase activity	22	> 100	+	$1.31*10^{-2}$
↳regulation of cysteine-type endopeptidase activity	27	> 100	+	$1.92*10^{-2}$
↳positive regulation of endopeptidase activity	26	> 100	+	$1.79*10^{-2}$
↳positive regulation of peptidase activity	26	> 100	+	$1.79*10^{-2}$
↳positive regulation of cellular protein metabolic process	234	69.18	+	$9.54*10^{-3}$
↳positive regulation of protein metabolic process	245	66.08	+	$1.09*10^{-2}$
↳positive regulation of catalytic activity	284	57.00	+	$1.69*10^{-2}$
↳positive regulation of molecular function	379	42.71	+	$3.99*10^{-2}$
↳regulation of cysteine-type endopeptidase activity involved in apoptotic process	24	> 100	+	$1.54*10^{-2}$
Unclassified	10416	.52	-	0.00
**PANTHER GO-Slim Biological Process** **(Genes downregulated after 24 hours)**	***Rattus norvegicus* (REF)**	**Fold-enrichment**	**+/-**	**P value**
transforming growth factor beta receptor signalling pathway	31	61.44	+	$3.78*10^{-2}$
↳enzyme linked receptor protein signalling pathway	302	16.82	+	$3.95*10^{-2}$
↳cell surface receptor signalling pathway	931	6.82	+	$2.16*10^{-3}$

↳signal transduction	2227	3.71	+	$3.21*10^{-2}$
↳cellular response to transforming growth factor beta stimulus	31	61.44	+	$3.78*10^{-2}$
↳response to transforming growth factor beta	31	61.44	+	$3.78*10^{-2}$
↳response to growth factor	138	23.00	+	$5.18*10^{-3}$
↳cellular response to growth factor stimulus	138	23.00	+	$5.18*10^{-3}$
positive regulation of phosphorylation	181	24.55	+	$2.73*10^{-5}$
↳regulation of phosphorylation	252	20.15	+	$1.00*10^{-5}$
↳regulation of phosphate metabolic process	276	18.40	+	$2.00*10^{-5}$
↳regulation of phosphorus metabolic process	276	18.40	+	$2.00*10^{-5}$
↳positive regulation of phosphate metabolic process	188	23.64	+	$3.51*10^{-5}$
↳positive regulation of phosphorus metabolic process	188	23.64	+	$3.51*10^{-5}$
regulation of cell population proliferation	173	22.02	+	$6.05*10^{-4}$
cell population proliferation	192	19.84	+	$1.09*10^{-3}$
transmembrane receptor protein tyrosine kinase signalling pathway	196	16.20	+	$2.72*10^{-2}$
regulation of protein phosphorylation	199	15.95	+	$2.92*10^{-2}$
protein phosphorylation	425	10.46	+	$7.52*10^{-3}$
↳phosphorylation	523	12.14	+	$1.08*10^{-5}$
↳phosphate-containing compound metabolic process	978	6.49	+	$3.36*10^{-3}$
↳phosphorus metabolic process	982	6.46	+	$3.49*10^{-3}$
multicellular organism development	935	6.11	+	$2.00*10^{-2}$
Unclassified	10416	.61	-	0.00
**PANTHER GO-Slim Cellular Component** **(Genes upregulated after 24 hours)**	***Rattus norvegicus*** **(REF)**	**Fold-enrichment**	**+/-**	**P value**

Unclassified	10266	0.53	-	0.00
**PANTHER GO-Slim Cellular Component** **(Genes downregulated after 24 hours)**	***Rattus norvegicus* (REF)**	**Fold-enrichment**	**+/-**	**P value**
Receptor complex	204	.32	+	$7.86*10^{-3}$
Unclassified	10266	0.53	-	0.00
**PANTHER GO-Slim Molecular function** **(Genes upregulated after 24 hours)**	***Rattus norvegicus* (REF)**	**Fold-enrichment**	**+/-**	**P value**
cysteine-type endopeptidase activity involved in apoptotic process	204	> 100	+	$1.77*10^{-3}$
Unclassified	11165	0.48	-	0.00
**PANTHER GO-Slim Molecular function** **(Genes downregulated after 24 hours)**	***Rattus norvegicus* (REF)**	**Fold-enrichment**	**+/-**	**P value**
growth factor binding	33	57.71	+	$1.24*10^{-2}$
transmembrane receptor protein tyrosine kinase activity	54	35.27	+	$4.96*10^{-2}$
↳transmembrane receptor protein kinase activity	67	37.90	+	$2.32*10^{-3}$
↳signalling receptor activity	1633	5.44	+	$3.45*10^{-5}$
↳molecular transducer activity	1633	5.44	+	$3.45*10^{-5}$
cytokine receptor binding	120	21.16	+	$2.11*10^{-2}$
receptor ligand activity	248	12.80	+	$2.25*10^{-2}$
↳signalling receptor activator activity	251	12.65	+	$2.38*10^{-2}$
↳signalling receptor regulator activity	272	11.67	+	$3.45*10^{-2}$
Unclassified	11165	.57	-	0.00
**PANTHER Pathways** **(Genes upregulated after 24 hours)**	***Rattus norvegicus* (REF)**	**Fold-enrichment**	**+/-**	**P value**

FAS signalling pathway	33	> 100	+	$2.35*10^{-3}$
Apoptosis signalling pathway	134	80.54	+	$3.61*10^{-2}$
Unclassified	18975	.57	-	0.00
**PANTHER Pathways** **(Genes downregulated after 24 hours)**	***Rattus norvegicus*** **(REF)**	**Fold-enrichment**	**+/-**	**P value**
Angiogenesis	174	21.89	+	$5.23*10^{-5}$
Unclassified	18975	.57	-	0.00
