# Supplementary figures and images for: Medium- and time-related effects on hypothermic storage of rat testicular cells
Source: Reprod Fertil. 2023 Jun 8;4(2):e220050. doi: 10.1530/RAF-22-0050 (PMC10305459; doi:10.1530/RAF-22-0050)

Supplementary figure 1

DMEM - GL (12h)

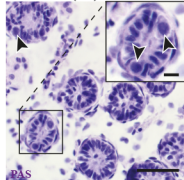

DMEM + GL (12h)

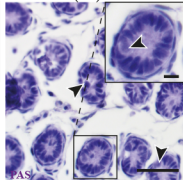

DMEM + GM (12h)

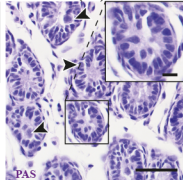

1xPBS (12h)

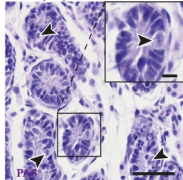

DMEM/F12 (12h)

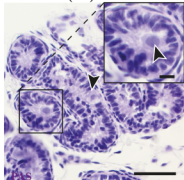

F12 (12h)

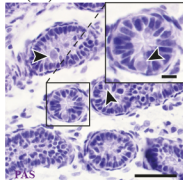

MEM (12h)

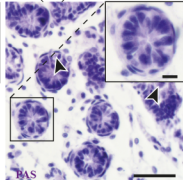

Fresh tissue control (0h)

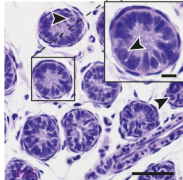

Supplement: Supplementary Figure 1 [file supplementary_figure_1.pdf]

Supplementary figure 2

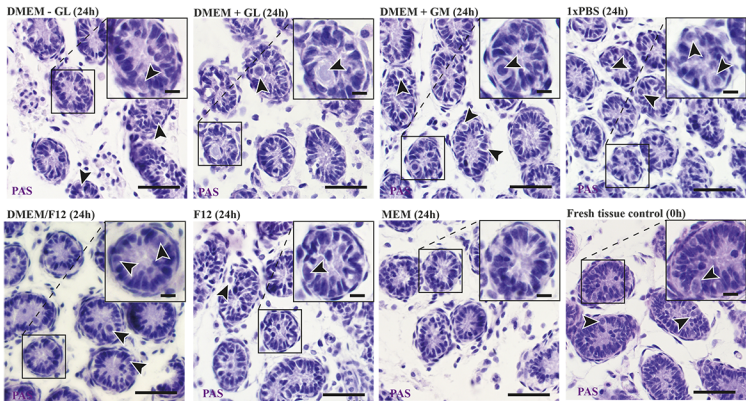

Supplement: Supplementary Figure 2 [file supplementary_figure_2.pdf]

Supplementary figure 3

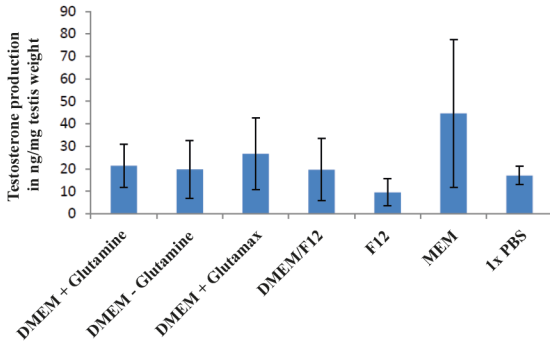

Supplement: Supplementary Figure 3 [file supplementary_figure_3.pdf]

Supplementary figure 4

# Effect of different media on germ cells gene profile

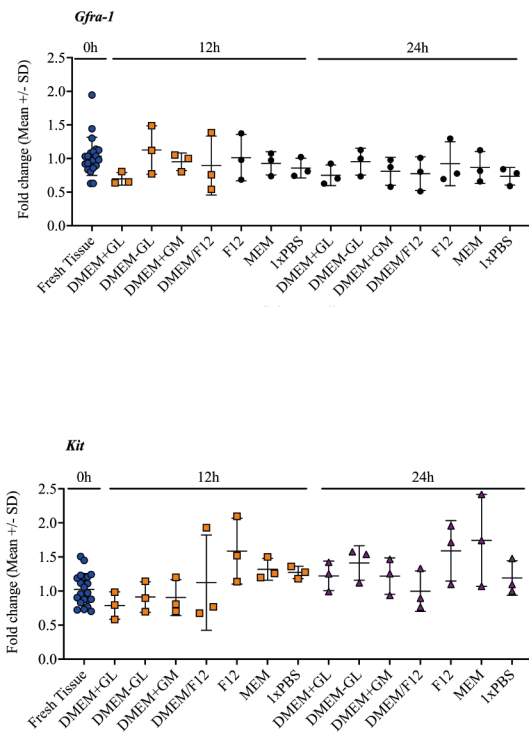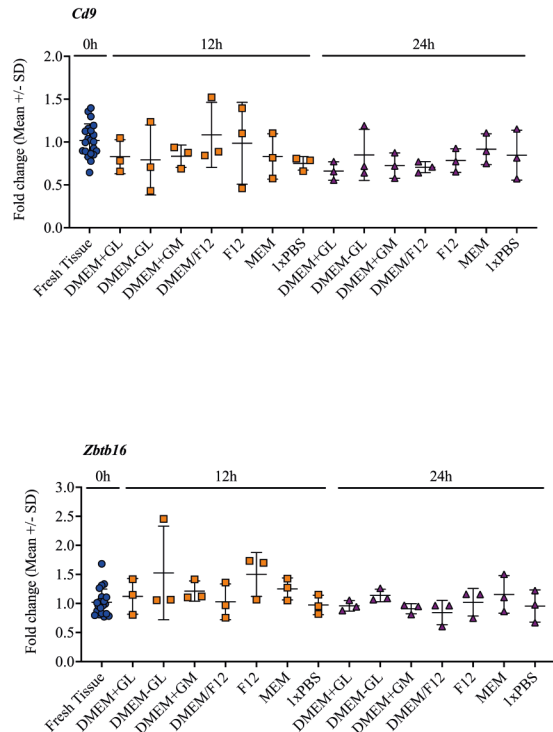

Supplement: Supplementary Figure 4 [file supplementary_figure_4.pdf]

Supplementary figure 5

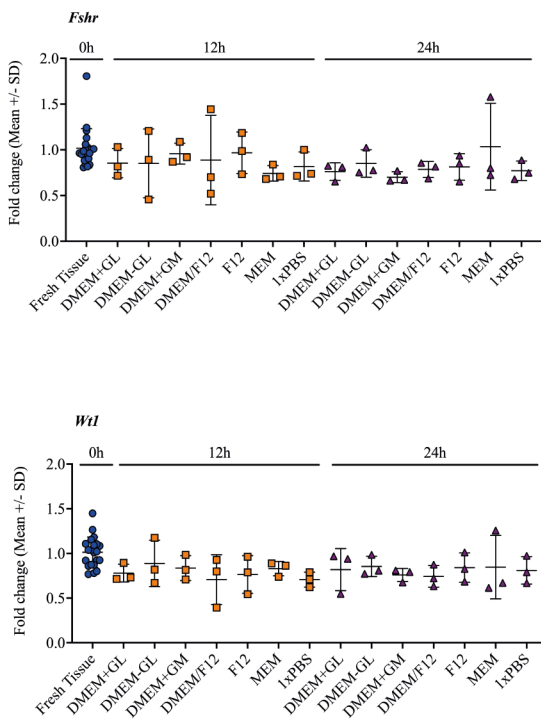

Effect of different media on Sertoli cells gene profile

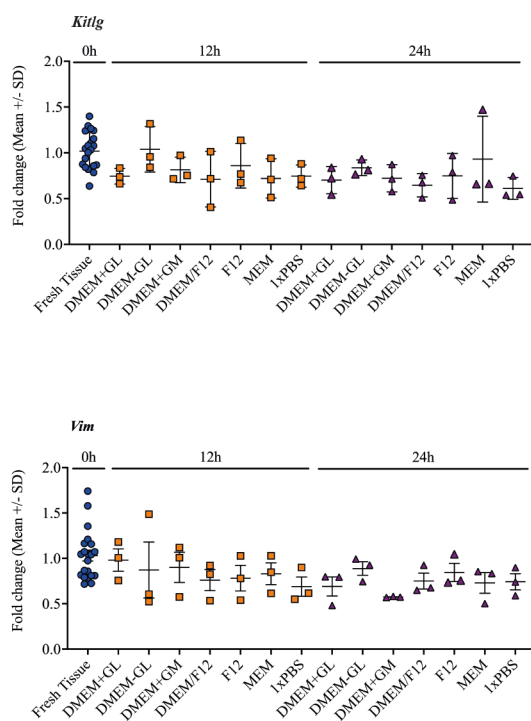

Supplement: Supplementary Figure 5 [file supplementary_figure_5.pdf]

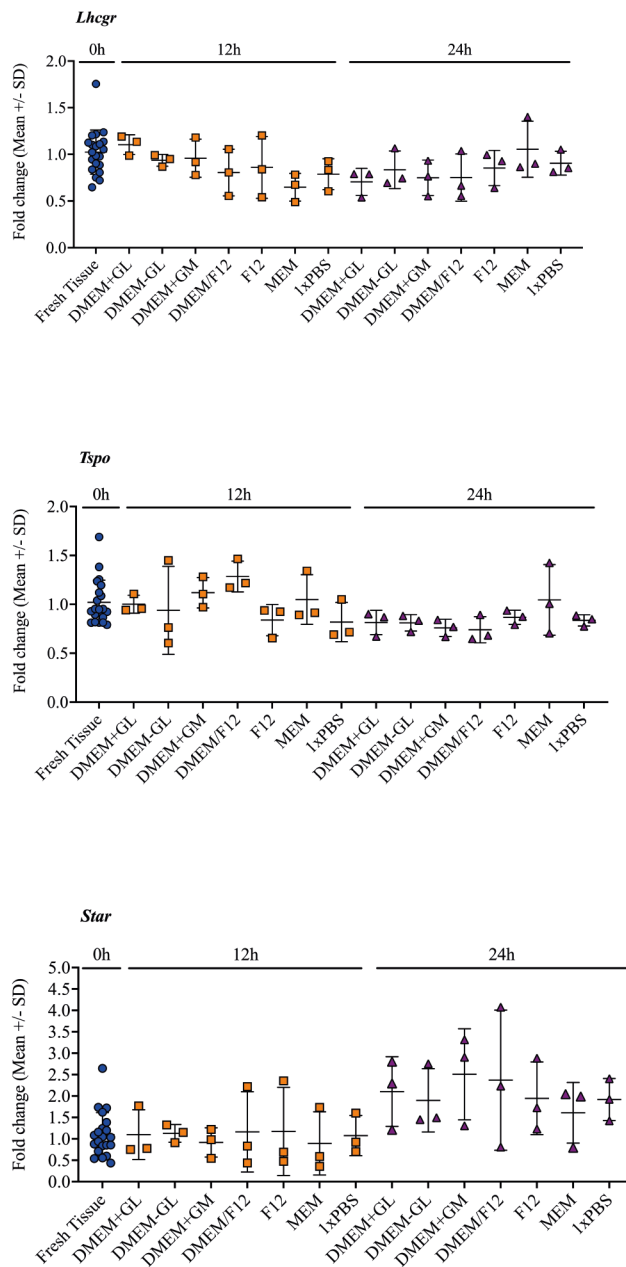

Supplement: Supplementary Figure 6 [file supplementary_figure_6.pdf]

Supplementary figure 7

Effect of different media on proliferation gene profile

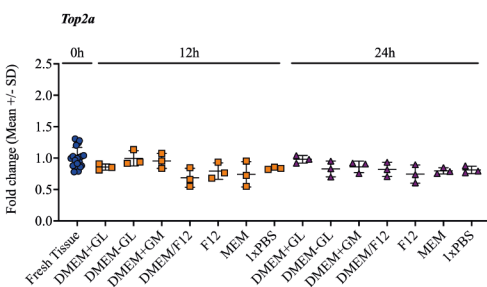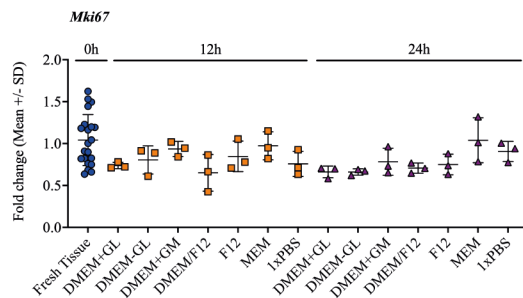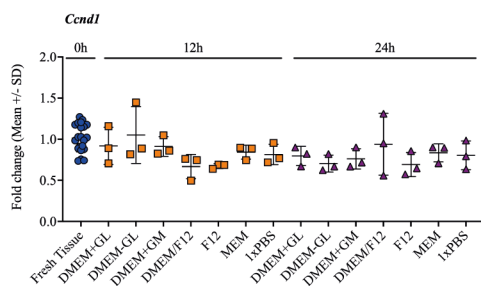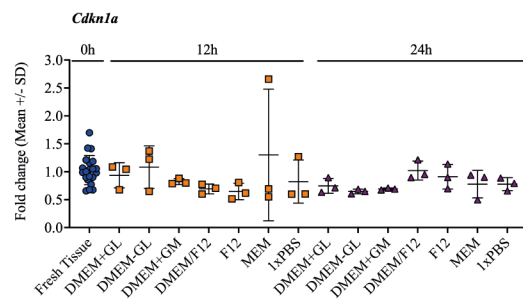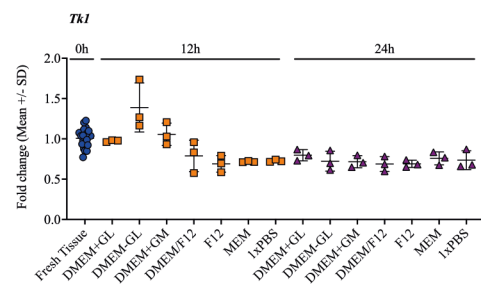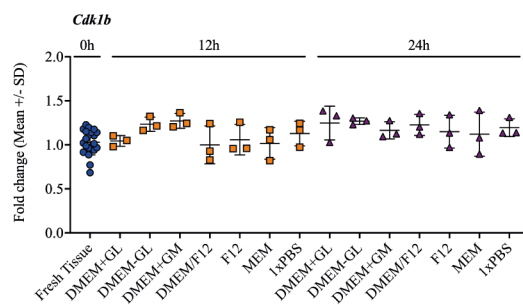

Supplement: Supplementary Figure 7 [file supplementary_figure_7.pdf]

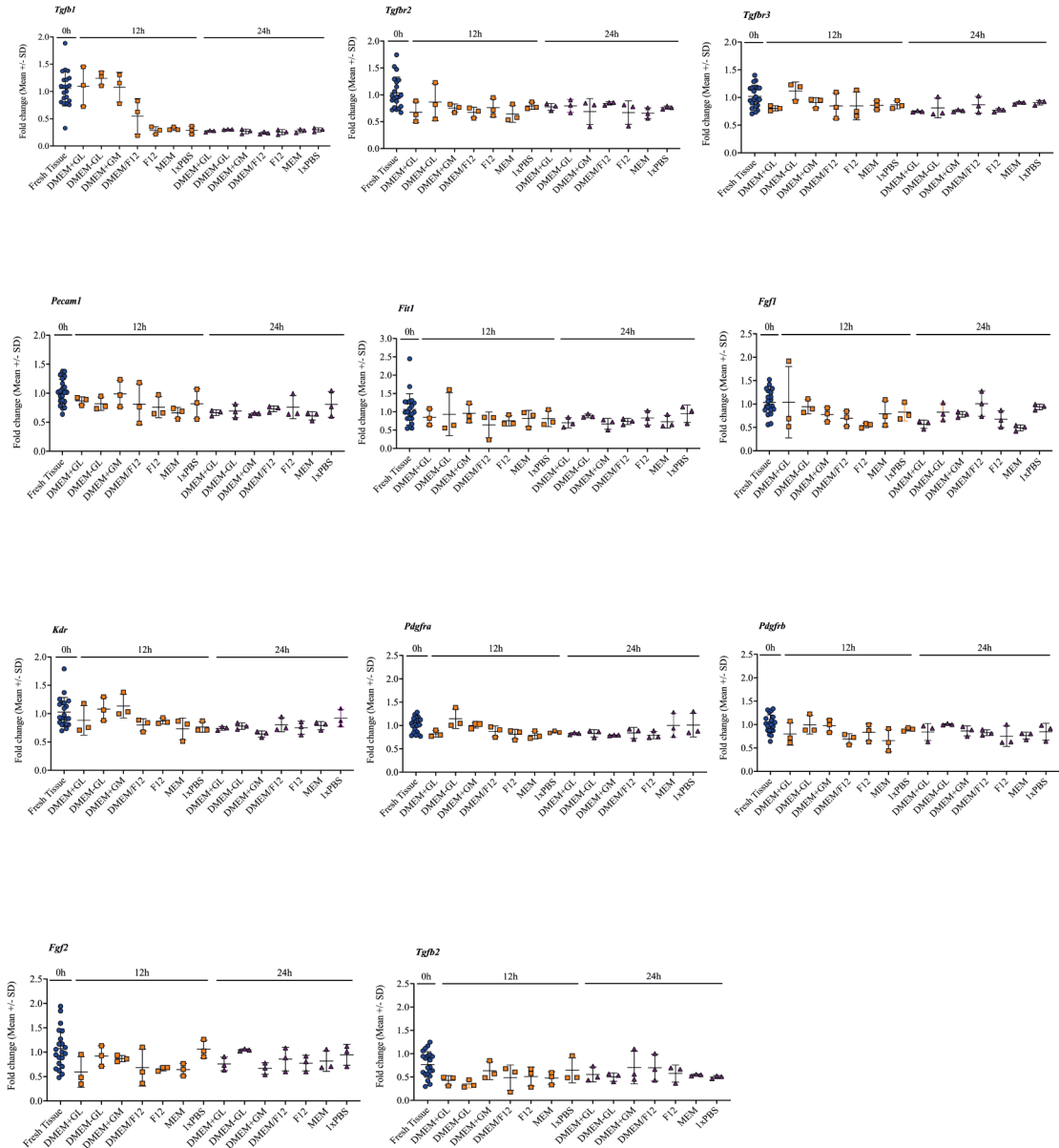

Supplement: Supplementary Figure 8 [file supplementary_figure_8.pdf]

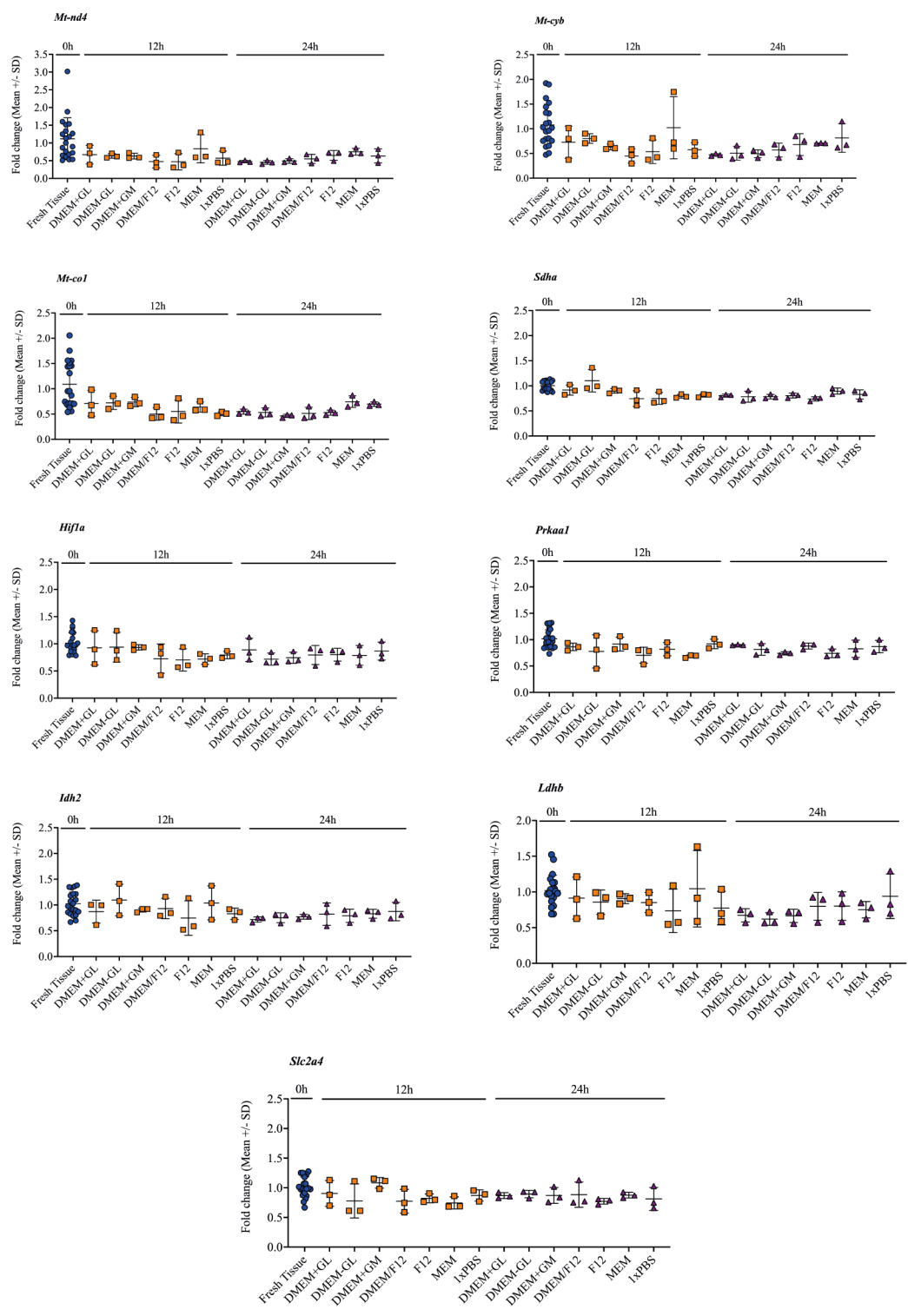

Supplement: Supplementary Figure 9 [file supplementary_figure_9.pdf]

Supplementary figure 10

Effect of different media on apoptotic gene profile

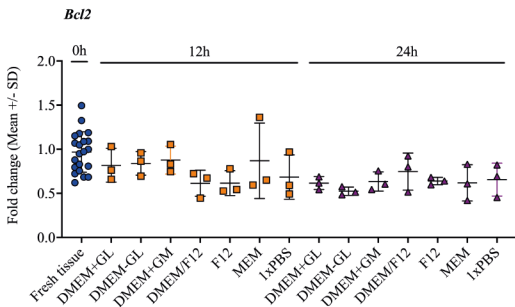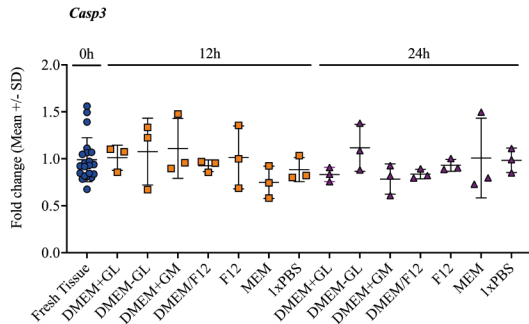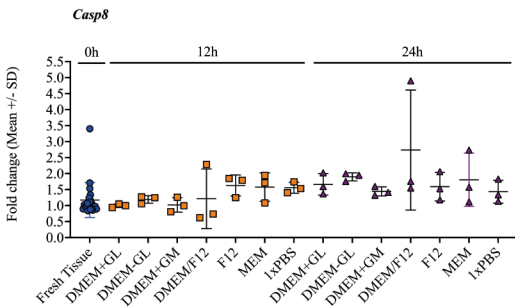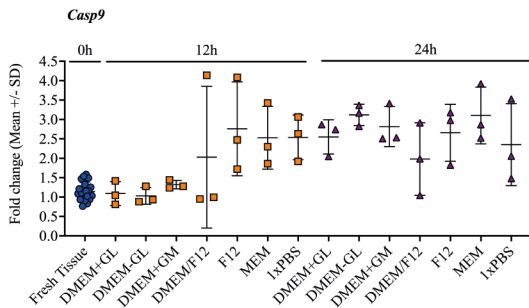

Supplement: Supplementary Figure 10 [file supplementary_figure_10.pdf]

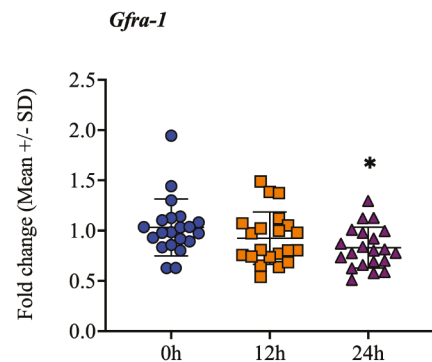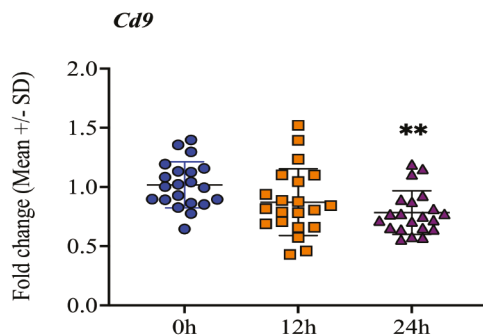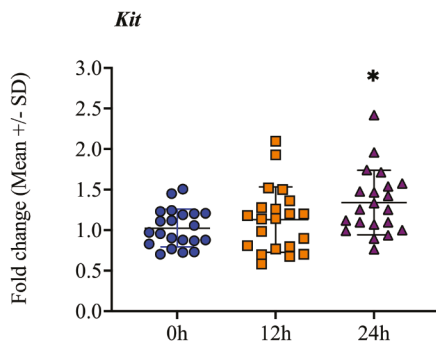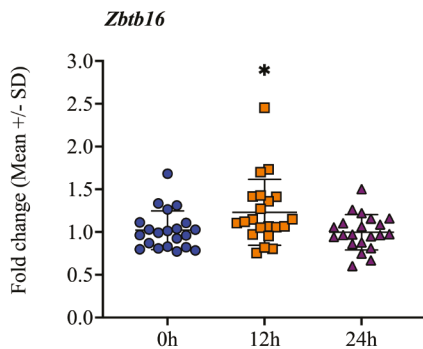

Supplement: Supplementary Figure 11 [file supplementary_figure_11.pdf]

Sertoli cell gene profile

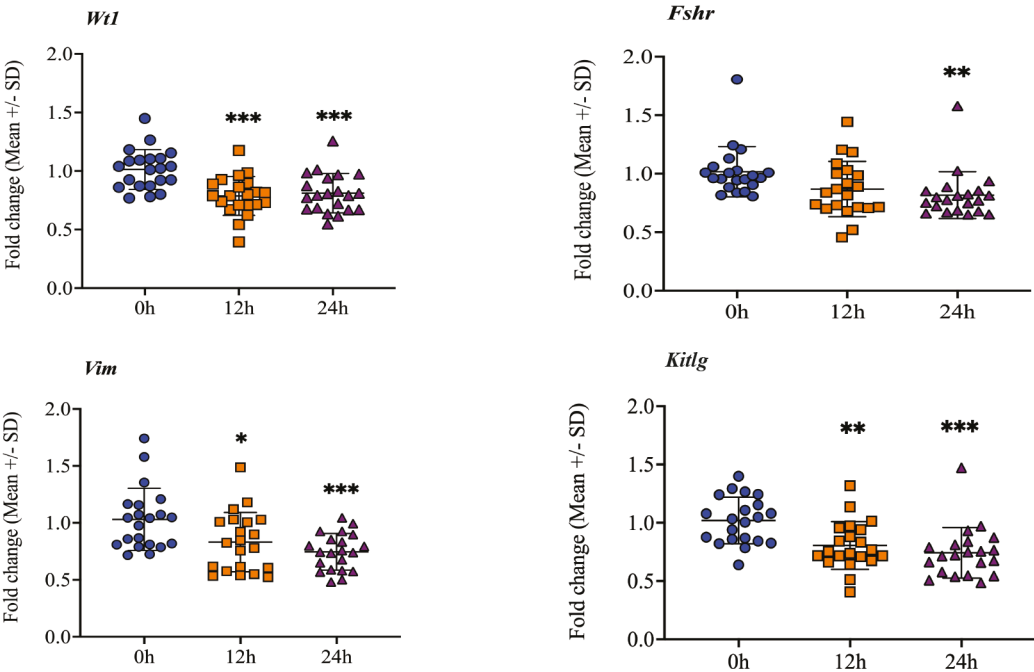

Leydig cell gene profile

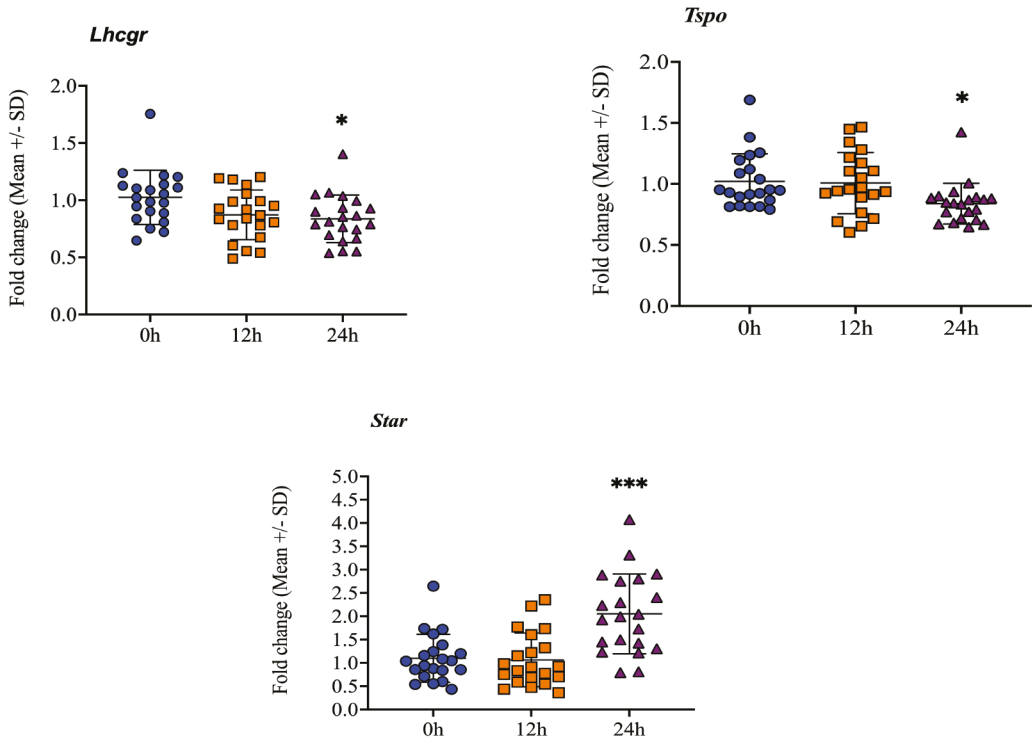

Supplement: Supplementary Figure 12 [file supplementary_figure_12.pdf]

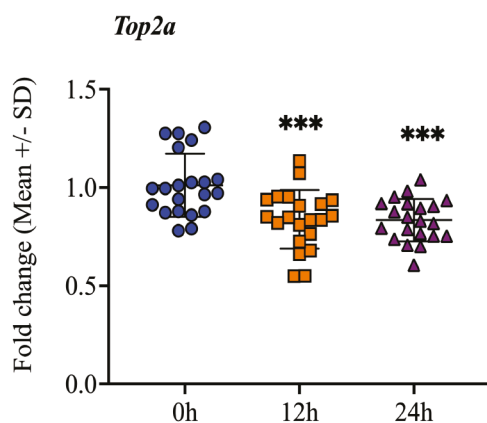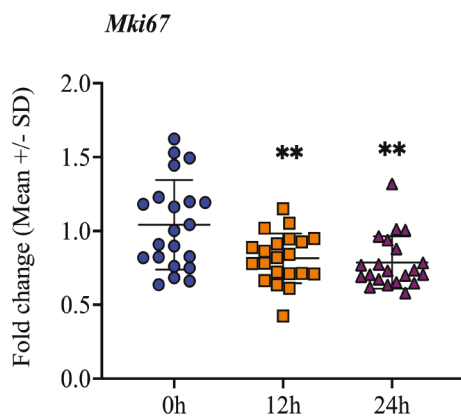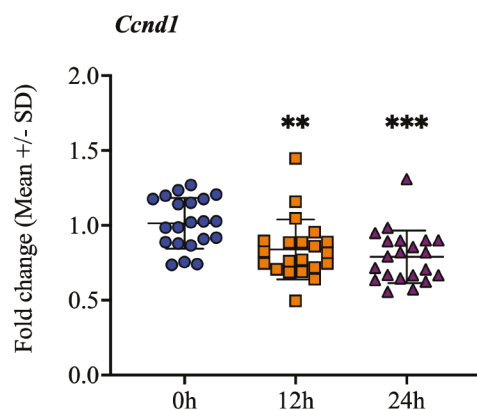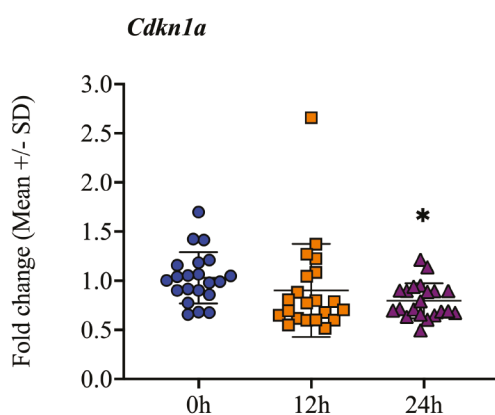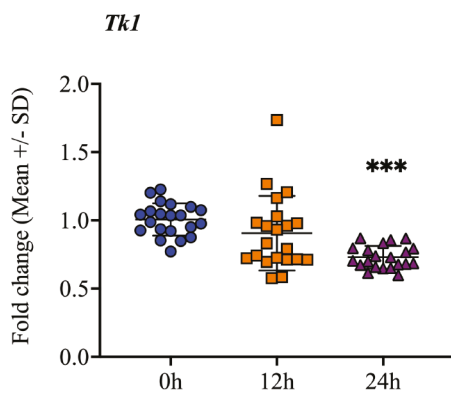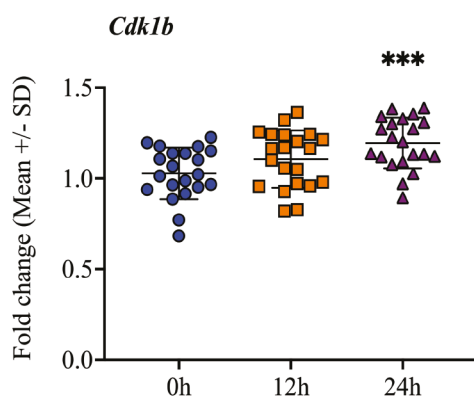

Supplement: Supplementary Figure 13 [file supplementary_figure_13.pdf]

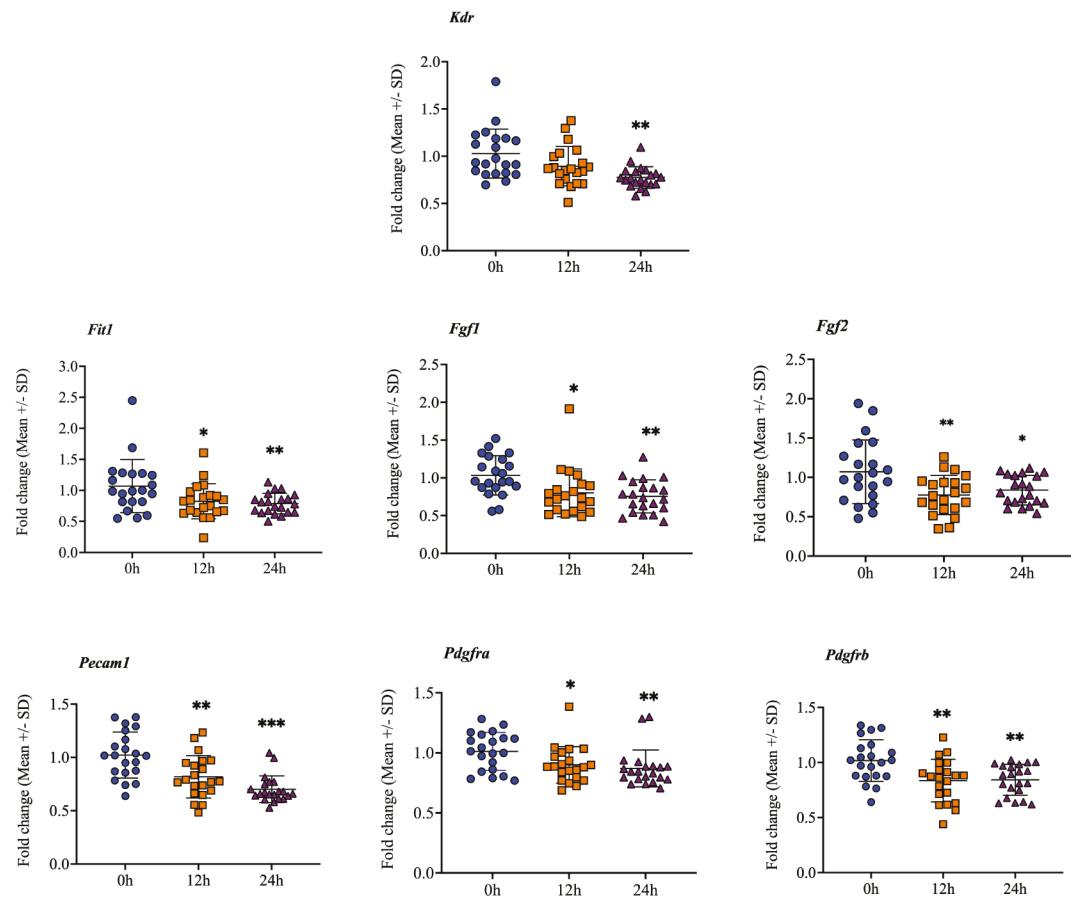

Supplement: Supplementary Figure 14 [file supplementary_figure_14.pdf]

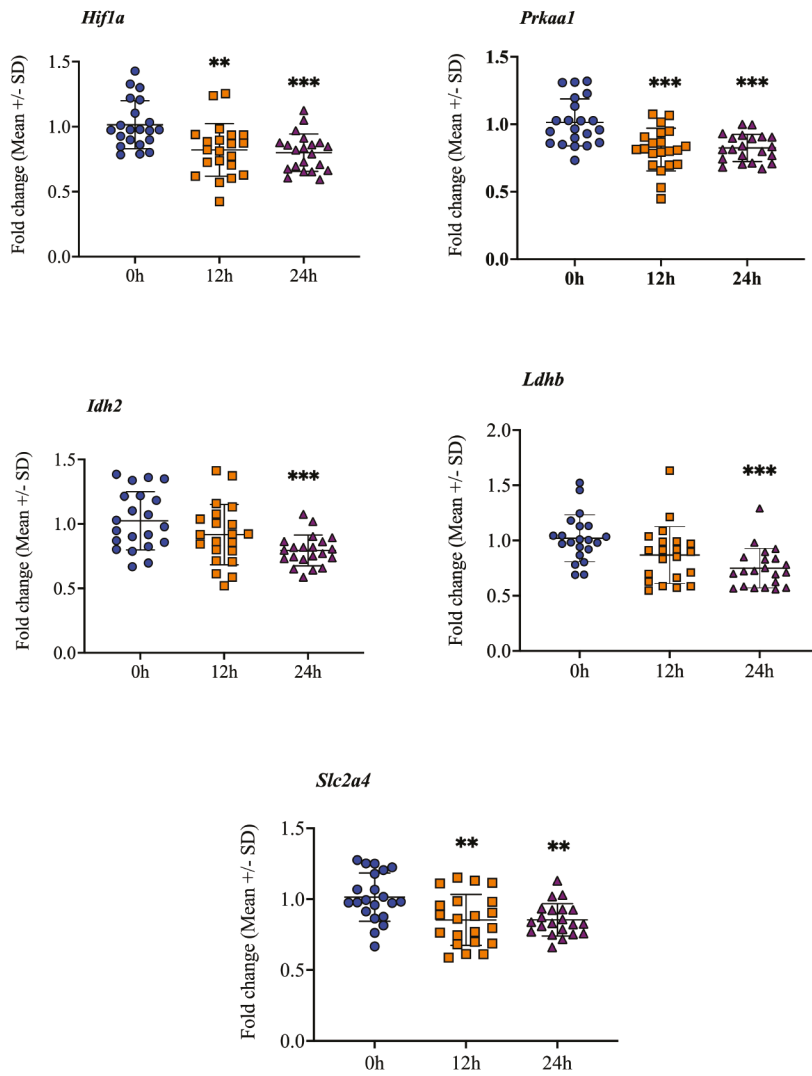

Supplement: Supplementary Figure 15 [file supplementary_figure_15.pdf]
